# Sarcoma: Molecular Pathology, Diagnostics, and Therapeutics

**DOI:** 10.3390/ijms24065833

**Published:** 2023-03-19

**Authors:** Shinji Miwa, Norio Yamamoto, Hiroyuki Tsuchiya

**Affiliations:** Department of Orthopaedic Surgery, Kanazawa University Graduate School of Medical Sciences, Kanazawa 920-8640, Japan

Although the incidence of sarcomas accounts for less than 1% of all malignancies, they are classified into more than 50 different subtypes with different biological behaviours. Due to their diversity and rarity, they present problems such as low diagnostic accuracy, limited treatment options, and difficulty in conducting clinical trials. To improve the clinical outcomes of sarcomas, advances in their management are required. This Special Issue includes the latest studies and reviews on the mechanisms of malignant transformation, molecular diagnosis, promising therapeutic targets, radiosensitisers, and combinations of anticancer drugs in various types of sarcoma.

Benini et al. investigated the diagnostic utility of molecular analysis compared with histological assessment in 2705 sarcoma tissue samples [1]. In this study, qRT–PCR and FISH analyses were conclusive in 74% and 76% of samples, respectively, whereas a combination of the methods was conclusive in 96% and 89% of frozen and formalin-fixed, paraffin-embedded tissues, respectively. The accurate prediction of sarcoma subtypes may be possible using a panel of different subtype-specific FISH probes and qRT–PCR assays.

This Special Issue includes two original studies and one review on rhabdomyosarcoma (RMS), the most common soft tissue sarcoma in childhood and adolescence. RMS is an aggressive disease, and there is no standard therapy for relapsed or advanced RMS. Approximately 25% of RMSs express fusion oncoproteins such as PAX3/PAX7-FOXO1, while fusion-negative RMS harbours RAS mutations. Radiotherapy plays an important role in local control in the treatment of RMS. Histone deacetylases have been identified as therapeutic targets in several tumour types, and histone deacetylases radiosensitise several types of cancer. Cassandri et al. evaluated the efficacy of the histone deacetylase inhibitor MS-275 (entinostat), in combination with radiation therapy, in combating RMS cells in vitro and in vivo [2]. The combination treatment inhibited DNA damage repair; increased reactive oxygen species formation; down-regulated NRF2, SOD, CAT, and GPx4 anti-oxidant genes; and induced G2 arrest. Based on these results, MS-275 is considered a promising agent for the treatment of RMS. Hindi et al. retrospectively investigated the activity of the alternative platinum-based regimen BOMP-EPI (bleomycin, vincristine, methotrexate, and cisplatin alternated with etoposide, cisplatin and ifosfamide) in adult patients with relapsed/metastatic RMS [3]. The best RECIST response (a complete response) occurred in 1/10 (10%) patients, a partial response in 5/10 (50%), stable disease in 3/10 (30%), and progression in 1/10 (10%). Since the outcome was superior to what was expected for this poor-prognosis population, BOMP-EPI is thought to be a promising chemotherapy regimen in adult patients with relapsed/metastatic RMS. Pomella et al. reviewed radiation therapy and possible radiosensitising strategies in patients with RMS [4]. In their article, radiosensitiser agents improved the cytotoxic effect of radiation therapy by enhancing the induction of DNA damage and the production of reactive oxygen species. The mechanisms of action mostly involve the enhancement of DNA damage due to the inhibition of DNA repair pathways, the inhibition of cell cycle progression, and the alteration of gene expression for resistance and sensitivity to IR. Further investigations are required on the topics of radioresistance mechanisms, effective radiosensitising strategies, the dosage and timing of radiation therapy, and radiosensitisers.

This Special Issue includes two articles that focus on the malignant transformation and progression of peripheral nerve tumours. Park et al. observed an increased expression of EGFR and a decreased expression of neurofibromin in MPNST and MPNST cells [5]. The silencing of NF1 expression following NF1 siRNA treatment or NF1-GAP-related domain overexpression demonstrated that EGFR expression levels were closely and inversely correlated with neurofibromin levels. Furthermore, the knockdown of NF1 following siRNA treatment augmented the nuclear localisation of phosphorylated SP1 (pSP1) and enhanced the binding of pSP1 to the EGFR gene promoter region. Their results suggest that EGFR expression may be upregulated by the activated Ras/ERK/SP1 signalling pathway, which can be enhanced by neurofibromin deficiency in NF1-associated MPNST. Kohlmeyer et al. investigated the mechanisms of the aberrant proliferation of Schwann cells, which are normally quiescent cells of the peripheral nervous system [6]. They discovered a new driver of malignant peripheral nerve sheath tumours, named RABL6A, which inhibits the tumour suppressor RB1. In their study, the induction of replicative senescence was associated with the reduced expression of RABL6A. Senescence is caused by RABL6A silencing in low-passage Schwann cells. RABL6A-deficient mouse embryonic fibroblasts (MEFs) showed impaired proliferation and accelerated senescence compared to wild-type MEFs. These results indicate that RABL6A is required for the maintenance of normal Schwann cell proliferation and suggest that malignant transformation may be promoted by aberrantly high RABL6A expression.

Several new anticancer drugs have recently been shown to exert antitumour effects in advanced sarcomas. Among the new anticancer drugs, eribulin, an anti-microtubule agent, has demonstrated antitumour effects in patients with L-sarcoma. In this Special Issue, López-Álvarez et al. demonstrated the efficacy of eribulin combined with gemcitabine in preclinical models of L-sarcoma [7]. In L-sarcoma cell lines, the combination of eribulin and gemcitabine was shown to have synergistic effects, increasing the number of hypodiploid events and the accumulation of DNA damage. In patient-derived xenograft models of L-sarcomas, eribulin combined with gemcitabine delayed tumour growth and was more effective in treating leiomyosarcoma. Based on these results, the combination of eribulin and gemcitabine is thought to be synergistic in L-sarcoma treatment.

Lopes et al. reviewed Kaposi’s sarcoma-associated herpesvirus (KSHV), known as human γ-herpesvirus 8 (HHV-8) [8]. HHV-8 modulates various cellular functions, including differentiation, proliferation, apoptosis, and survival. In their review, they described important KSHV genes that can induce malignant characteristics of Kaposi’s sarcoma. 

Osteosarcoma is the most common malignant bone neoplasm in children, adolescents, and young adults. Although various tumour markers and therapeutic targets have been reported in different malignancies in recent years, there are no useful molecular markers or therapeutic targets for the management of osteosarcoma. Pekarek et al. reviewed their risk factors and biomarkers, including their clinical uses, prognoses, and limitations [9].

Chondrosarcoma, the second most common primary bone sarcoma, is characterised by the production of cartilaginous matrix and accounts for approximately 30% of malignant bone tumours. There are limited treatment options for patients with metastatic or unresectable chondrosarcomas. Recent basic and clinical studies have investigated the efficacy and safety of new treatment modalities for advanced chondrosarcomas. Miwa et al. reviewed recent studies on gene mutations, biomarkers, anticancer agents, immunotherapy, and other promising treatments for chondrosarcomas [10]. Recent studies have suggested several promising biomarkers and therapeutic targets for chondrosarcomas, including IDH1/2, COL2A1, and PD-L1. In addition, several molecular targeting agents and immunotherapies have shown favourable antitumour activity in clinical studies of patients with advanced chondrosarcoma. 

In summary, this Special Issue of the *International Journal of Molecular Sciences* presents a collection of articles that discuss the latest basic and clinical studies on sarcomas. Although there are limited treatment options for sarcoma patients, recent studies have demonstrated promising therapeutic targets, anticancer agents, and combinations of these treatments. The articles presented in this Special Issue may help improve the management of sarcomas. We greatly appreciate the contributions of the authors of the articles published in this Special Issue. 

## References

[1] Benini S., Gamberi G., Cocchi S., Magagnoli G., Fortunato A.R., Sciulli E., Righi A., Gambarotti M. (2022). The Efficacy of Molecular Analysis in the Diagnosis of Bone and Soft Tissue Sarcoma: A 15-Year Mono-Institutional Study. Int. J. Mol. Sci..

[2] Cassandri M., Pomella S., Rossetti A., Petragnano F., Milazzo L., Vulcano F., Camero S., Codenotti S., Cicchetti F., Maggio R. (2021). MS-275 (Entinostat) Promotes Radio-Sensitivity in PAX3-FOXO1 Rhabdomyosarcoma Cells. Int. J. Mol. Sci..

[3] Hindi N., Carrillo-Garcia J., Blanco-Alcaina E., Renshaw M., Luna P., Duran J., Jimenez N., Sancho P., Ramos R., Moura D.S. (2023). Platinum-Based Regimens Are Active in Advanced Pediatric-Type Rhabdomyosarcoma in Adults and Depending on HMGB1 Expression. Int. J. Mol. Sci..

[4] Pomella S., Porrazzo A., Cassandri M., Camero S., Codenotti S., Milazzo L., Vulcano F., Barillari G., Cenci G., Marchese C. (2022). Translational Implications for Radiosensitizing Strategies in Rhabdomyosarcoma. Int. J. Mol. Sci..

[5] Park G.H., Lee S.J., Lee C.G., Kim J., Park E., Jeong S.Y. (2021). Neurofibromin Deficiency Causes Epidermal Growth Factor Receptor Upregulation through the Activation of Ras/ERK/SP1 Signaling Pathway in Neurofibromatosis Type 1-Associated Malignant Peripheral Nerve Sheet Tumor. Int. J. Mol. Sci..

[6] Kohlmeyer J.L., Kaemmer C.A., Umesalma S., Gourronc F.A., Klingelhutz A.J., Quelle D.E. (2021). RABL6A Regulates Schwann Cell Senescence in an RB1-Dependent Manner. Int. J. Mol. Sci..

[7] Lopez-Alvarez M., Gonzalez-Aguilera C., Moura D.S., Sanchez-Bustos P., Mondaza-Hernandez J.L., Martin-Ruiz M., Renshaw M., Ramos R., Castilla C., Blanco-Alcaina E. (2022). Efficacy of Eribulin Plus Gemcitabine Combination in L-Sarcomas. Int. J. Mol. Sci..

[8] Lopes A.O., Marinho P.D.N., Medeiros L.D.S., de Paula V.S. (2022). Human Gammaherpesvirus 8 Oncogenes Associated with Kaposi’s Sarcoma. Int. J. Mol. Sci..

[9] Pekarek L., De la Torre-Escuredo B., Fraile-Martinez O., Garcia-Montero C., Saez M.A., Cobo-Prieto D., Guijarro L.G., Saz J.V., De Castro-Martinez P., Torres-Carranza D. (2022). Towards the Search for Potential Biomarkers in Osteosarcoma: State-of-the-Art and Translational Expectations. Int. J. Mol. Sci..

[10] Miwa S., Yamamoto N., Hayashi K., Takeuchi A., Igarashi K., Tsuchiya H. (2022). Therapeutic Targets and Emerging Treatments in Advanced Chondrosarcoma. Int. J. Mol. Sci..

